# Internet Addiction Management: A Comprehensive Review of Clinical Interventions and Modalities

**DOI:** 10.7759/cureus.55466

**Published:** 2024-03-04

**Authors:** Yatika Chadha, Ragini Patil, Saket Toshniwal, Nayan Sinha

**Affiliations:** 1 Psychiatry, Jawaharlal Nehru Medical College, Datta Meghe Institute of Higher Education and Research, Wardha, IND; 2 Medicine, Jawaharlal Nehru Medical College, Datta Meghe Institute of Higher Education and Research, Wardha, IND

**Keywords:** ethical considerations, relapse prevention, psychotherapeutic approaches, diagnostic criteria, clinical interventions, internet addiction

## Abstract

Internet addiction is a pervasive and complex issue that has gained increasing attention in the digital age. This comprehensive review provides an in-depth exploration of clinical interventions and modalities for managing internet addiction. It begins by examining the diagnostic criteria and assessment tools used to identify internet addiction, highlighting the diverse subtypes and varying degrees of severity. Subsequently, the review delves into various clinical interventions, including psychotherapeutic approaches like cognitive behavioral therapy (CBT), dialectical behavior therapy (DBT), and mindfulness-based interventions. Pharmacological interventions, technology-based tools, and integrative approaches are also thoroughly analyzed. The review also outlines various treatment settings and modalities such as inpatient treatment centers, outpatient clinics, telehealth, support groups, and prevention programs for schools and communities. Furthermore, it discusses the efficacy and challenges associated with managing internet addiction, emphasizing the need for effective interventions, relapse prevention, ethical considerations, and addressing stigma and access barriers. In conclusion, the review offers practical implications for clinical practice. It emphasizes future research's importance in refining diagnostic criteria, exploring emerging technologies, and adapting interventions to an ever-evolving digital landscape. This comprehensive review is a valuable resource for clinicians, researchers, and policymakers seeking to understand and address the complexities of internet addiction.

## Introduction and background

In an era of unprecedented digital connectivity, the internet has become an integral part of our daily lives, revolutionizing how we communicate, work, learn, and entertain ourselves. However, the ubiquity of the internet has brought to light a concerning issue - internet addiction. Internet addiction, often referred to as problematic or compulsive internet use, is a growing behavioral disorder that affects individuals across diverse age groups, cultures, and backgrounds [1]. This complex phenomenon is marked by an uncontrollable and excessive preoccupation with online activities, leading to negative consequences in various facets of life, including physical and mental health, relationships, and overall well-being [2].

As the digital landscape continues to evolve, understanding and addressing internet addiction has become imperative for healthcare professionals, researchers, policymakers, and individuals and families impacted by this condition. This comprehensive review delves into the multifaceted realm of internet addiction management, providing an in-depth exploration of clinical interventions, treatment modalities, ethical and legal considerations, and emerging developments in the field [3].

Throughout this review, we will navigate through the diagnostic criteria and assessment tools used to identify internet addiction, explore the intricacies of internet addiction subtypes and severity, and shed light on co-occurring disorders that often accompany this condition. We will unravel the therapeutic approaches clinicians employ, such as cognitive behavioral therapy (CBT) and mindfulness-based interventions, along with pharmacological and technology-based interventions that hold promise in treating internet addiction. Furthermore, we will delve into the diverse settings and modalities for treatment, ranging from inpatient centers to telehealth options and community prevention programs.

## Review

Clinical assessment of internet addiction

Diagnostic Criteria and Assessment Tools

Problematic internet use (PIU) criteria: These criteria parallel the diagnostic criteria commonly used for substance use disorders. They encompass features such as unsuccessful attempts to reduce internet use, cravings, and withdrawal symptoms when not using the internet. Much like substance addiction, individuals with PIU may find it challenging to control their online behaviors. They may experience intense cravings to return to online activities, even when they wish to reduce internet use. The presence of withdrawal symptoms when not using the internet suggests that the behavior has become ingrained and potentially addictive, leading to discomfort or distress when abstaining from it [4].

Compulsive internet use criteria: This set of criteria hones in on the compulsive nature of internet use, highlighting elements such as preoccupation with the internet, loss of control over use, and neglect of other activities. Preoccupation refers to obsessive thoughts about the internet, which can interfere with an individual's ability to focus on other aspects of life. The loss of control manifests as an inability to limit the time spent online or to refrain from certain online activities. Neglect of other activities signifies that an individual may prioritize online activities to the detriment of essential daily responsibilities and interests. Together, these criteria capture the essence of compulsivity, a core feature of addiction [5].

Negative consequences criteria: These criteria focus on the adverse consequences of internet use, including problems in work or school, strained relationships, and emotional distress. The negative consequences of internet addiction can be far-reaching, affecting an individual's academic or professional life, personal relationships, and emotional well-being. These criteria underline the impact of internet addiction on an individual's daily functioning and overall quality of life. They demonstrate how PIU extends beyond mere recreational activities and can impair essential domains of an individual's existence [3].

Internet addiction test (IAT): Developed by Dr. Kimberly Young, the IAT is a widely recognized and utilized self-report questionnaire designed to assess the extent of an individual's internet addiction and its impact on their daily life. The IAT typically consists of statements or questions about an individual's internet usage habits. Respondents rate the degree to which each statement applies to them, often on a scale from one to five, with higher scores indicating a greater level of internet addiction. The IAT covers various aspects of internet use, including time spent online, consequences in daily life, and emotional well-being. It provides a quantitative measure to help clinicians and researchers gauge the severity of internet addiction, making it a valuable tool for diagnosis and treatment planning [6].

Compulsive internet use scale (CIUS): The CIUS is another self-report questionnaire designed to measure the severity of compulsive internet use. It is a shorter and more focused assessment tool, primarily concentrating on the compulsive aspect of internet addiction. The CIUS assesses features such as preoccupation with internet use, loss of control over online behavior, withdrawal symptoms when not using the internet, and the impact of internet use on daily life and relationships. The scale provides a quantifiable measure of the level of compulsion associated with internet addiction, making it a valuable tool for diagnosing and assessing specific aspects of this condition [7].

Young's diagnostic questionnaire (YDQ): Dr. Kimberly’s YDQ is a concise and user-friendly assessment tool consisting of eight yes-or-no questions. It serves as a quick screening tool to identify PIU and potential signs of internet addiction. While it is less comprehensive than the IAT or CIUS, the YDQ is valuable for its simplicity and ease of administration, making it a useful initial screening tool in various clinical and research settings. The questions in the YDQ cover core features of internet addiction such as preoccupation, withdrawal symptoms, and neglect of other activities [8].

Identifying Subtypes and Severity of Internet Addiction

Types of internet use: Recognizing the various subtypes of internet addiction, such as online gaming addiction, social media addiction, or pornography addiction, is pivotal for effective treatment. Different types of internet use may elicit distinct behavioral patterns and underlying motivations. Identifying the specific type of addiction enables clinicians to tailor treatment strategies to address the unique challenges associated with each subtype. For instance, treatment for online gaming addiction might involve strategies to reduce excessive gaming time and develop healthier leisure activities. In contrast, treatment for social media addiction could focus on addressing the emotional aspects of overuse and enhancing social skills [9].

Severity levels: Internet addiction can manifest with varying degrees of severity, from mild to severe. Clinicians frequently use rating scales to assess the severity of an individual's addiction. These scales consider factors such as the amount of time spent online, the degree of impairment in daily life, and the presence of withdrawal symptoms. Determining the severity level of internet addiction is essential for treatment planning. It helps clinicians prioritize interventions, allocate appropriate resources, and establish realistic treatment goals. More severe cases may require more intensive treatment, while milder cases may benefit from outpatient or self-help strategies [10].

Patterns and triggers: Understanding an individual's patterns of internet use and identifying triggers for excessive use is crucial for treatment planning and relapse prevention. Patterns of use may reveal specific times, situations, or emotional states that trigger excessive internet use. Recognizing these patterns allows clinicians to help individuals develop coping strategies to manage cravings and prevent relapse. Additionally, pinpointing triggers can guide individuals in making lifestyle changes and building self-awareness, empowering them to make healthier choices and reducing the risk of returning to PIU [11].

Co-occurring Disorders and Comorbidities

Depression and anxiety: It is not uncommon for individuals with internet addiction to experience symptoms of depression and anxiety. The relationship between these mental health conditions and internet addiction is complex, as excessive internet use can lead to social isolation, reduced physical activity, and disrupted sleep patterns, all of which can contribute to the development or exacerbation of mood and anxiety disorders. On the other hand, individuals with pre-existing depression and anxiety may turn to the internet as a coping mechanism, seeking solace or distraction from their symptoms [12].

Substance use disorders: A noteworthy overlap exists between internet addiction and substance use disorders. Some individuals may resort to substances, such as drugs or alcohol, as a means of self-medication or distraction from the negative consequences of their internet addiction. Substance use can further complicate the clinical picture, as it introduces a dual diagnosis situation. Treating both internet addiction and substance use disorders concurrently requires an integrated approach that addresses both issues [13].

Attention-deficit/hyperactivity disorder (ADHD): Individuals with ADHD may be at higher risk of developing internet addiction due to their inherent impulsivity and difficulty in self-regulation. The instant gratification and constant stimulation offered by the internet can be particularly appealing to those with ADHD, making it challenging for them to moderate their internet use. Recognizing this co-occurrence is vital for tailoring interventions and strategies that address both ADHD and internet addiction simultaneously [14].

Eating disorders: Internet use, especially on social media platforms and image-focused websites, can exacerbate or co-occur with eating disorders such as anorexia or bulimia. The pressure to conform to societal standards of beauty and body image on these platforms can trigger or worsen disordered eating behaviors. Additionally, excessive time spent online may disrupt eating routines and physical activities, contributing to the development of eating disorders. Treating individuals with co-occurring internet addiction and eating disorders requires a comprehensive approach that addresses body image issues, self-esteem, and online influences [15].

Clinical interventions

Psychotherapeutic Approaches

CBT: CBT is a cornerstone of therapeutic approaches for internet addiction and is widely used. It is based on the premise that an individual's thoughts, feelings, and behaviors are interconnected. In the context of internet addiction, CBT helps individuals identify maladaptive thought patterns and behaviors related to excessive internet use. Individuals learn to challenge and change negative thought patterns and impulsive behaviors through cognitive restructuring and modification. CBT equips them with coping strategies and self-regulation skills, enabling them to gain better control over their online behavior. This efficient approach empowers individuals to make conscious, healthier choices regarding their internet use [16].

Dialectical behavior therapy (DBT): DBT, rooted in cognitive-behavioral techniques, combines mindfulness and acceptance strategies. It is particularly effective for individuals with internet addiction who struggle with emotional dysregulation and impulsive behavior. DBT teaches clients distress tolerance, emotional awareness, and effective emotion regulation. These skills are essential for managing the emotional triggers that can lead to excessive internet use. By enhancing emotional control and resilience, DBT equips individuals with the tools they need to address the underlying emotional issues that may be driving their addictive behavior [17].

Mindfulness-based interventions: Mindfulness practices, such as meditation and mindfulness-based cognitive therapy, are invaluable for treating internet addiction. These practices foster self-awareness by helping individuals become more attuned to their thoughts, emotions, and behaviors. Mindfulness encourages individuals to be present at the moment, which can aid in controlling impulsive online behaviors and promoting self-regulation. Mindfulness-based interventions are beneficial for developing a healthier relationship with the internet by increasing awareness and enabling individuals to make conscious choices about their online activities [18].

Family and group therapy: Internet addiction often extends beyond the individual, impacting their family and social relationships. Family therapy and group therapy sessions offer a supportive and educational environment for both the individual with addiction and their loved ones. These sessions serve multiple purposes, including addressing communication issues, setting healthy boundaries, and fostering mutual understanding. Family and group therapy also provide a network of support and encouragement, which can be instrumental in the recovery process. The involvement of loved ones in therapy is essential for creating a conducive home environment that supports the individual's recovery [19].

Pharmacological Interventions

Medications for co-occurring disorders: Individuals with internet addiction often experience co-occurring mental health conditions such as depression, anxiety, or ADHD. The treatment plan may involve prescribing medications to target these underlying issues in such cases. For instance, antidepressants may be recommended for those with comorbid depression, while anxiolytics can help manage anxiety symptoms. By addressing these co-occurring disorders, individuals may experience a reduction in the emotional distress and symptoms that contribute to their internet addiction. Medication can complement other therapeutic interventions and support overall mental well-being [20].

Medications targeting internet addiction: While there are no specific medications approved for the treatment of internet addiction, ongoing research explores the potential use of certain medications to reduce cravings and compulsive behaviors related to excessive internet use. One example is naltrexone, which is commonly used to treat substance addictions. Some studies suggest that naltrexone might also be effective in reducing cravings associated with internet addiction, although more research is needed to establish its efficacy and safety for this purpose. The use of medications targeting internet addiction is an evolving area of study and may provide additional options for treatment in the future [21].

Technology-Based Interventions

Internet blocking and filtering software: Internet blocking and filtering software are valuable tools against internet addiction. These applications enable individuals to restrict access to specific websites or apps, helping reduce the temptation to engage in PIU. By blocking access to time-wasting or addictive websites, individuals can create digital barriers that support their self-control efforts. Such software is often part of a broader treatment plan, complementing therapeutic approaches. While they are not a standalone solution, these tools serve as a helpful aid in curbing excessive internet use and promoting healthier online habits [3].

Digital detox programs: Digital detox programs are structured interventions designed to help individuals temporarily disconnect from the internet and reset their relationship with technology. These programs typically involve periods of abstinence from the internet, allowing individuals to step away from screens and the digital world. Following a period of disconnection, digital detox programs guide individuals through a gradual reintegration process, focusing on promoting healthy and mindful use of technology. By providing a structured framework for reducing internet use, digital detox programs support individuals in regaining control over their online behaviors and developing a more balanced approach to technology [22].

Smartphone apps for self-control: The availability of smartphone apps designed for self-control and screen time management has grown significantly. These apps empower users to track their screen time, set usage limits, and receive real-time feedback on their digital habits. Users can customize their goals and restrictions, helping them stay accountable for their online behavior. These apps often include features like app blocking, usage history tracking, and notifications to remind users of their goals. Smartphone apps for self-control offer individuals a practical and accessible way to monitor and regulate their screen time, fostering a more balanced and intentional use of digital devices [23].

Combined and Integrative Approaches

Personalized treatment plans: Integrative treatment plans for internet addiction aim to tailor interventions to the individual's needs and challenges. By conducting a thorough assessment, clinicians can identify the most pressing issues and determine the most effective interventions for the person in question. This personalized approach ensures that treatment is not one-size-fits-all but adapts to each individual's unique circumstances [24].

Holistic address of the issue: Internet addiction often involves various interconnected factors, including behavioral patterns, emotional triggers, family dynamics, and technological influences. Integrative treatment combines multiple interventions such as CBT for behavior modification, family therapy for addressing interpersonal dynamics, and internet-blocking software for practical management. This holistic approach enables a more comprehensive understanding of addiction and allows clinicians to address all relevant aspects effectively [25].

Comprehensive support: Internet addiction can have a profound impact on an individual's life and their loved ones. Integrative treatment provides comprehensive support not only for the person with the addiction but also for their family and social network. This ensures that the individuals in the support system understand the nature of the addiction, how to foster a supportive environment, and how to establish healthy boundaries [26].

Flexibility and adaptability: Internet addiction can manifest in various ways, and its contributing factors can change over time. Integrative approaches are flexible and adaptable, allowing for adjustments to the treatment plan as the individual progresses and new challenges arise. This flexibility ensures that the treatment remains practical and relevant throughout recovery [3].

Combined expertise: Integrative treatment programs often involve a team of professionals with expertise in different areas such as psychology, family therapy, and technology. This collaborative effort brings diverse skills and knowledge to the treatment process, enhancing its effectiveness [27].

Modalities and settings

Inpatient Treatment Centers

Detoxification: In some severe cases of internet addiction, individuals may require a digital detox to break the cycle of excessive use. Inpatient treatment centers can provide a controlled environment where individuals are removed from access to the internet and digital devices. This period of detox allows individuals to experience a break from their addiction, gain perspective, and begin to develop healthier habits. During this phase, the focus is on withdrawal from the internet, emotional regulation, and developing coping strategies for life without constant online engagement [28].

Therapeutic programming: Inpatient treatment programs offer a structured and supportive environment that includes individual therapy, group therapy, and a range of therapeutic modalities to address both the addiction and underlying psychological issues. These therapeutic sessions help individuals explore the root causes of their addiction, develop coping strategies, and build essential life skills. Group therapy provides opportunities for peer support and shared experiences, enhancing recovery. Therapeutic programming in inpatient settings is integral to addressing addiction comprehensively and promoting long-term recovery [29].

Crisis intervention: For individuals who are at immediate risk of self-harm or harm to others due to their internet addiction, inpatient treatment centers offer crisis intervention. In a controlled and supervised environment, individuals receive the urgent care and support they need to ensure their safety. Crisis intervention involves risk assessment, safety planning, and close monitoring to address the immediate threats posed by the addiction. The goal is to stabilize the individual and provide them with the necessary resources to address their addiction more effectively [30].

Medication management: Inpatient treatment centers can provide close oversight for individuals who are prescribed medication as part of their treatment plan. This level of care ensures that medications are taken as prescribed and that any potential side effects or adverse reactions are closely monitored. Medication management in inpatient settings is precious for individuals with co-occurring mental health conditions, as it allows for coordinated and integrated care that addresses both addiction and underlying psychiatric disorders [31].

Outpatient Clinics

Individual and group therapy: Outpatient therapy is a cornerstone of treatment for internet addiction. It often includes evidence-based therapeutic modalities such as CBT, which helps individuals identify and modify maladaptive thought patterns and behaviors related to excessive internet use. Group therapy sessions allow individuals to connect with peers facing similar challenges. Sharing experiences and coping strategies in a supportive group setting can be a powerful component of recovery. Individual therapy provides a personalized approach, allowing clinicians to address each client's unique needs and circumstances [32].

Flexibility: Outpatient care allows individuals to continue their daily routines, including work or school commitments. This is especially beneficial for those who need to maintain their responsibilities and cannot commit to an inpatient program. Outpatient treatment allows individuals to integrate the strategies and skills learned in therapy into their everyday lives, promoting real-world application and sustainable change [33].

Family involvement: The involvement of family members can be an integral part of outpatient treatment. Family therapy sessions provide the opportunity to engage the individual's support network in the recovery process. Family therapy can address communication issues, set boundaries, and foster understanding, creating a more supportive home environment. The involvement of loved ones not only aids in the individual's recovery but also helps family members better understand and cope with the challenges of internet addiction [19].

Telehealth and Online Therapy

Video conferencing: Telehealth platforms provide a secure and effective means for therapists and clients to engage in therapy sessions through video calls. Video conferencing allows face-to-face interaction, fostering a more personal and connected therapeutic experience. It enables individuals to access treatment without needing physical travel, making it particularly beneficial for those with busy schedules or living in remote areas. Video-based therapy sessions offer the advantages of real-time communication, visual cues, and building a strong therapeutic relationship with the clinician [34].

Chat-based therapy: Some telehealth platforms offer text-based therapy for individuals who prefer written communication. Chat-based therapy allows individuals to exchange messages with their therapist in a secure and private online environment. This mode of therapy is ideal for those who feel more comfortable expressing themselves in writing or prefer asynchronous communication. Chat-based therapy provides flexibility regarding when and how individuals engage in therapeutic conversations [35].

Access to specialists: Telehealth expands access to specialized therapists and treatment options. Regardless of geographic location, individuals can connect with therapists who specialize in internet addiction or co-occurring mental health conditions. Telehealth eliminates the distance barrier, ensuring individuals have access to the expertise they need for effective treatment. This is especially advantageous for those seeking highly specialized care that may not be available locally [36].

Support Groups and Self-Help Resources

Twelve-step programs: Adaptations of traditional 12-step programs, such as Internet and Technology Addicts Anonymous, provide individuals with a structured support network. These programs follow a step-by-step approach to recovery, emphasizing self-awareness, personal responsibility, and peer support. Participating in a 12-step program can help individuals connect with others with similar struggles and experiences. It offers a sense of camaraderie, accountability, and a clear path toward recovery. These programs can be precious for individuals who prefer a structured, group-based approach to addressing their addiction [37].

Online forums and communities: Internet-based support groups and forums offer a sense of community and understanding for those struggling with internet addiction. These platforms allow individuals to share their experiences, challenges, and successes. They can seek advice, encouragement, and empathy from others who have faced similar issues. Online communities offer a level of anonymity, making it more comfortable for individuals to open up about their addiction. These platforms can be a valuable source of ongoing support and a way to connect with a broader network of people dealing with internet addiction [38].

Self-help books and workbooks: Self-help materials, including books and workbooks, can provide individuals with guidance, exercises, and strategies for managing their internet use. These resources often offer a structured approach to self-reflection and behavior modification. Individuals can work through these materials at their own pace and convenience, making them a flexible option for those who prefer a self-directed approach to recovery. Self-help materials are beneficial for individuals who are motivated to take control of their addiction and are seeking practical tools and insights [39].

Prevention Programs for Schools and Communities

Educational workshops: Schools often provide workshops and educational programs to teach students about responsible internet use. These programs can cover various topics, including digital literacy, online safety, and the signs of internet addiction. By educating students about the potential risks and helping them develop healthy online habits, schools can empower young individuals to make informed choices regarding their internet use. These workshops also provide a space for open discussions about the challenges and benefits of the digital world [40].

Parenting workshops: Programs designed for parents are invaluable in helping them understand and manage their children's internet use. These workshops provide parents with the knowledge and skills to navigate their children's online experiences, set boundaries, and support responsible internet use. Parenting workshops also address the importance of modeling healthy online behavior and maintaining open communication with their children. Equipping parents with the tools to guide their children's internet use fosters a supportive and responsible digital environment at home [41].

Community awareness campaigns: Public campaigns can raise awareness about the risks of internet addiction and provide resources for individuals and families. These campaigns inform the community about the signs of internet addiction, the potential consequences, and where to seek help. They also emphasize the importance of balanced technology use and digital wellness. Community awareness campaigns aim to reduce the stigma associated with internet addiction, encourage early intervention, and promote a culture of responsible internet use [26].

Efficacy and challenges

Effectiveness of Clinical Interventions

Research findings: Examining research findings entails a comprehensive review of studies and research outcomes focused on the effectiveness of various treatment modalities for internet addiction. This involves thoroughly assessing published research, clinical trials, and empirical studies exploring different treatment approaches' outcomes. Research findings can reveal which interventions have demonstrated significant positive results, the factors influencing their effectiveness, and any potential limitations. Such an analysis provides a basis for evidence-based decision-making in clinical practice, guiding therapists and clinicians in choosing the most suitable treatment strategies for individuals with internet addiction [42].

Outcome measures: The discussion of outcome measures in the context of internet addiction treatment involves an exploration of the metrics and criteria used to assess the success of clinical interventions. Outcome measures encompass a range of quantitative and qualitative assessments, such as changes in time spent online, improvements in emotional well-being, and enhanced social functioning. By examining the specific outcome measures employed in research and clinical practice, professionals can gain insights into the variables considered essential for evaluating the effectiveness of treatment. This discussion also highlights the importance of choosing appropriate, valid, and reliable outcome measures to gauge treatment progress accurately [43].

Treatment success rates: The summary of treatment success rates provides an overview of the achievements in reducing internet addiction and enhancing the quality of life for individuals undergoing treatment. This section typically presents data on the percentage of individuals who experienced significant improvements in their condition following specific treatment modalities. Success rates may vary depending on the treatment approach, the severity of the addiction, and the presence of co-occurring disorders. This summary offers valuable insights into the real-world impact of various treatment strategies and can inform clinicians and individuals seeking help about the likelihood of achieving positive outcomes. It also underscores the need for ongoing research and development of more effective interventions to improve treatment success rates [44].

Relapse Prevention and Long-Term Outcomes

Relapse factors: Recognizing and comprehensively addressing relapse factors is paramount in sustaining recovery from internet addiction. This involves identifying the triggers, situations, and psychological states that may lead to a return to PIU. Understanding these factors allows individuals to develop coping strategies and preventative measures. Relapse prevention plans typically involve strategies to manage stress, handle cravings, and identify early warning signs of relapse. Common relapse factors in internet addiction may include emotional distress, social isolation, and exposure to internet-related cues. By understanding these triggers, individuals can better navigate the challenges threatening their recovery [11].

Maintenance strategies: The discussion of maintenance strategies centers on exploring the tools and techniques that support long-term recovery. These strategies are designed to help individuals maintain the progress achieved during treatment and prevent relapse. Maintenance strategies often encompass ongoing self-monitoring, the cultivation of alternative leisure activities, time management skills, and the development of a balanced and fulfilling life offline. Additionally, individuals may benefit from continuing their therapeutic practices, such as mindfulness or cognitive-behavioral skills, to reinforce healthy habits. By proactively implementing maintenance strategies, individuals can reduce the risk of relapse and ensure a sustained and healthier relationship with the internet [45].

Follow-up and aftercare: The role of continued support and aftercare in sustaining recovery cannot be overstated. After completing initial treatment, individuals often benefit from follow-up and aftercare programs, including ongoing therapy sessions, support group participation, and check-ins with their treatment providers. Aftercare reinforces the skills and insights gained during treatment and offers a support network. This support is essential in addressing any challenges in the transition to independent recovery. Additionally, aftercare can provide a safety net for individuals facing stressful or triggering situations, reducing the likelihood of relapse [46].

Ethical and Legal Considerations

Confidentiality and privacy: Protecting individuals' privacy and data in the digital age is a fundamental ethical consideration. Treatment providers must uphold strict confidentiality standards to ensure that sensitive information about the individual's addiction, co-occurring conditions, and progress in treatment remains secure. This is particularly crucial in the digital realm, where data breaches and privacy concerns are prevalent. Ethical guidelines and legal regulations, such as the Health Insurance Portability and Accountability Act (HIPAA) in the United States, dictate treatment providers' steps to safeguard an individual's personal and treatment-related information. Individuals seeking treatment must have confidence in the confidentiality of their interactions with healthcare professionals [47].

Informed consent: Ethical issues surrounding informed consent are of great importance, especially when treating minors with internet addiction. Informed consent involves providing individuals, or their legal guardians in the case of minors, with comprehensive information about the nature of treatment, potential risks and benefits, and available alternatives. It is essential that individuals and their guardians fully understand the treatment process and willingly agree to participate. Treating minors requires careful consideration of their capacity to provide informed consent, and ethical guidelines dictate the involvement of guardians or legal representatives to make decisions in the minor's best interest [48].

Legal obligations: Treatment providers have legal responsibilities when managing internet addiction cases. These obligations may include mandatory reporting of child abuse or neglect, ensuring compliance with relevant laws and regulations, and acting in the best interests of the individual seeking treatment. Additionally, treatment providers may need to adhere to specific legal requirements for their professional licensing and practice. Understanding and adhering to these legal obligations is essential for ethical and effective treatment while protecting the rights and well-being of the individuals seeking care [49].

Stigma and Barriers to Treatment

Social stigma: The stigma associated with internet addiction can have a profound effect on help-seeking behavior. Individuals who struggle with internet addiction may fear judgment, ridicule, or social isolation if they disclose their condition. This fear of stigmatization can deter individuals from reaching out for support, even when they recognize the need for treatment. Addressing social stigma involves raising awareness, providing education, and fostering a more empathetic and understanding society. Reducing stigma can encourage individuals to seek help, promoting early intervention and recovery [50].

Cultural differences: Cultural attitudes and beliefs can significantly influence perceptions of internet addiction and attitudes toward treatment. In some cultures, mental health issues, including behavioral addictions like internet addiction, may carry different levels of acceptance or understanding. Cultural norms and values may impact whether individuals perceive their internet use as problematic and are open to seeking treatment. Clinicians and treatment providers must be culturally sensitive and adapt their approaches to align with their client's cultural backgrounds and beliefs. Additionally, cultural competency in mental health care is essential to ensure equitable access to treatment for individuals from diverse cultural backgrounds [51].

Access barriers: Several barriers can limit access to treatment for internet addiction, including a lack of awareness about the condition, financial constraints, and limited access to treatment resources. Many individuals may need to know that internet use has become problematic, which can delay help-seeking. Additionally, the cost of treatment and the availability of affordable options may deter individuals from accessing care. Limited access to treatment resources, particularly in underserved areas, can be a significant barrier. Addressing these access barriers involves community education, the development of affordable treatment options, and efforts to expand the availability of treatment resources in urban and rural areas [26].

Future Directions for Research and Practice

Emerging treatment approaches: As the understanding of internet addiction continues to evolve, new and innovative treatment approaches are being explored. These may include novel therapeutic techniques, digital interventions, or integrative approaches incorporating emerging technologies. For instance, virtual reality therapy, online support groups, or gamified interventions may play a role in future treatments. Understanding these emerging approaches is essential for staying at the forefront of effective treatment and addressing the ever-evolving challenges of internet addiction [52].

Technological advancements: Technology can be harnessed for prevention and intervention. New tools and applications are being developed to help individuals monitor and manage their internet use, set healthy boundaries, and access support resources. Technological advancements, such as artificial intelligence-driven chatbots, mobile apps, and wearable devices, offer innovative prevention and early intervention solutions. Additionally, telehealth platforms continue to advance, providing more accessible and effective means of delivering treatment. It is essential to stay informed about these technological advancements to enhance the field's capacity to address internet addiction [53].

Policy and regulation: The role of government, industry, and healthcare organizations in shaping policy and regulations related to internet addiction is crucial. Government bodies may implement regulations to address internet addiction as a public health concern. Industry stakeholders, including technology companies, can contribute to responsible design and promoting healthy technology use. Healthcare organizations can advocate for and influence policies that support access to treatment and insurance coverage for internet addiction. Understanding the evolving policy and regulation landscape is essential for promoting comprehensive and ethical management of internet addiction [54].

## Conclusions

In conclusion, this comprehensive review has shed light on the multifaceted nature of internet addiction, its prevalence, and its far-reaching impacts on individuals and society. We explored the various diagnostic criteria and assessment tools employed by clinicians to identify and classify internet addiction, recognizing its diverse subtypes and severity levels. The review delved into clinical interventions, encompassing psychotherapeutic approaches, pharmacological strategies, technology-based tools, and integrated methods. Moreover, we examined the array of treatment modalities and settings available, from inpatient treatment centers to online therapy and community-based prevention programs. As we assessed the efficacy and challenges of managing internet addiction, we emphasized the importance of research findings, the need for ongoing support to prevent relapse, and the ethical considerations in the treatment process. Addressing stigma and barriers to treatment was another critical aspect discussed. In conclusion, the implications for clinical practice point toward a patient-centered, evidence-based approach that adapts to individual needs while ensuring ethical and legal standards. Lastly, this review has underscored the necessity of future research, from refining diagnostic criteria to exploring the impact of emerging technologies, all of which will contribute to more effective and holistic strategies for managing internet addiction in an increasingly connected world.
